# Effects of spatial location and household wealth on health insurance subscription among women in Ghana

**DOI:** 10.1186/1472-6963-13-221

**Published:** 2013-06-17

**Authors:** Akwasi Kumi-Kyereme, Joshua Amo-Adjei

**Affiliations:** 1 Department of Population and Health, Faculty of Social Sciences, University of Cape Coast, Cape Coast, Ghana

**Keywords:** Health insurance, Ecological zones, Wealth quintile, Ghana

## Abstract

**Background:**

This study compares ownership of health insurance among Ghanaian women with respect to wealth status and spatial location. We explore the overarching research question by employing geographic and proxy means targeting through interactive analysis of wealth status and spatial issues.

**Methods:**

The paper draws on the 2008 Ghana Demographic and Health Survey. Bivariate descriptive analysis coupled with binary logistic regression estimation technique was used to analyse the data.

**Results:**

By wealth status, the likelihood of purchasing insurance was significantly higher among respondents from the middle, richer and richest households compared to the poorest (reference category) and these differences widened more profoundly in the Northern areas after interacting wealth with zone of residence. Among women at the bottom of household wealth (poorest and poorer), there were no statistically significant differences in insurance subscription in all the areas.

**Conclusions:**

The results underscore the relevance of geographic and proxy means targeting in identifying populations who may be need of special interventions as part of the efforts to increase enrolment as well as means of social protection against the vulnerable.

## Background

In developing countries, the cost of health care financing is one of the biggest social challenges and out-of-pocket payments present significant drains on household spending patterns, making health insurance one of the best social interventions [1]. However, health financing is challenged by a number of problems such as public under-funding, high user fees and informal payments, causing the poor to be deprived of health care [2].

In Ghana, the legal framework for mandatory health insurance was promulgated in 2004 through an Act of Parliament, Act 650. While the establishment of an insurance scheme was in fulfilment of political promise made by the then ruling government during the 2000 general elections campaign, it was more of a response to a need to improve equity and access to health services in the face of increasing cost of health care.

The WHO [3] concept of equity in health financing states that individuals must be given the needed service based on the ability to pay. This therefore calls for a system of financing that pools risks and resources from a greater majority to protect the most vulnerable against cost of illness [4-6]. Thus, making health insurance in poor countries a reflection of social justice [7].

In spite of the perceived successes of the health insurance scheme in Ghana, especially in terms of the number of subscribers, the major objective of achieving equity is believed to be failing, given the low enrolment of the poor [8-10]. Unhealthy poor people, especially women, stand a higher risk of being denied access to health care because of their ability to afford. Equity in health insurance is therefore intended to avoid preclusion of people who are vulnerable due to their socioeconomic conditions as well as health states [10].

A number of theoretical propositions have been expounded in the health insurance literature to explain decision-making processes towards subscriptions to health insurance in poor settings. Schneider [11] provided one of such seminal reviews of theoretical paradigms on the subject. Among the variants of decision making models Schneider [11] espoused are consumer choice, expected utility, state-dependent utility, prospect, cumulative prospect, endowment/status quo/veil of experience, regret and disappointment, time preferences, poverty and social capital. Whereas all the decision making models provide us with several intuitive explanations to why people buy or do not buy insurance, the time preferences model, the state-dependent utility model and the poverty model help us to examine the influence of certain socioeconomic and demographic variables on insurance subscription among Ghanaian women. In this regard, this paper is nested within theoretical triangulation [12], thus examining the data from multiple-perspectives. Time preference decision model is posited around present and future assessment of health needs based on present circumstances. Thus, if people value future than present consumptions, current consumption could be deferred for investments in the future, which could be a decision to be insured whereas preference for the present could constrain deferring present consumption for the future. According to the World Bank [13] cited in Schneider [11], those with high future time preference would purchase insurance. The state-dependent utility approach assumes that individuals engage in introspection of their subjective health state before purchasing health insurance. If an individual considers his/her risk of illness to be low, there is less likelihood of purchasing insurance. On the other hand, if perceived risk of illness is high, a person would subscribe to an insurance provider. The poverty position of Schneider’s decision making is simply concerned with ability to pay high premiums. When individuals and households consider premiums to be out of their reach, then they are unlikely to be insured.

From spatial perspective, geographic targeting (GT) [9] provides a useful strategy for identifying people who need insurance most based on physical location. Geographic targeting is a population level measure more than individual level measure. It is often premised on the need to identify populations who reside in specific locations with comparatively higher levels of poverty. GT is often combined with welfare or wealth status measures as a way of identifying populations whose needs are not being served adequately [9]. Although a useful conceptual construct for identifying the poor for interventions, GT can also lead to outflows to the rich, who are usually not the targets for interventions [14-16].

This paper contributes to the debate on purchasing of health insurance by examining the interactive effect of spatial location and household wealth status with special emphasis on women. Our focus on women is justified by the fact that this population, in the context of a developing nation, faces more health challenges than men, particularly as a result of reproductive health risks. By this, we do not seek to negate the health needs of men; just that women constitute a special population in our context. A recent study by Mensah et al. [17] provides us with additional good reasons to focus on women. Mensah and his colleagues found that, between insured and uninsured women, those insured reported better reproductive health outcomes than the uninsured and that the achievement of the Millennium Development Goals could be accelerated by up-scaling health insurance coverage. Our emphasis on women in the reproductive ages (15–49) will therefore enable us to highlight the characteristics of the women who could be targeted for interventions. Apart from the population of interest, it is only the study by Akazili et al. [1], to the best of our knowledge, which used a nationally representative data (Ghana Living Standards Survey-2005). All the other studies we identified did not use nationally representative samples [6-8,17-19]. Our use of a nationally representative data therefore widens the scope in analysing the characteristics of insured and uninsured women.

This study proceeds on the assumption that there are no significant interactive effects of household wealth status and spatial location on health insurance subscription among women in Ghana. Yet we are also mindful of the fact that there are other hypothetically pertinent dynamics which influence whether an individual would insure or remain uninsured. With respect to our dataset, the following variables: education, age, religion, partner’s education, autonomy (determined by health decision making, large and small household purchases, see [20]) are controlled for in our analysis.

## Methods

### Context

Ghana is one of the first countries in sub-Saharan Africa to introduce a nation-wide health insurance scheme with an intention of replacing out-of-pocket payment health care regime. Prior to the state-sponsored implementation of health insurance in the country, the Catholic Church, through one of its hospitals in the Nkoranza District of the Brong-Ahafo Region, operated an insurance scheme, albeit limited to in-patient care. Fulfilling political campaign promise, the New Patriotic Party introduced an insurance scheme in 2004 following parliamentary approval. The scheme is financed with funds from a National Health Insurance (NHI) levy, collected from an additional 2.5% on value-added tax (VAT); a monthly deduction of 2.5% from each formal sector worker’s contribution to the Social Security and National Insurance Trust (SSNIT) pension fund; interests from investments made by the scheme; an annual premium contribution from all informal sector workers and those formal sector workers who are not covered by the SSNIT pension scheme; and a registration fee paid by all National Health Insurance Scheme (NHIS) subscribers to their respective District Mutual Health Insurance Schemes (DMHIS) with which they are enrolled in the scheme. While the premiums are preferred to be charged on ability to pay, the absence of reliable income data of several Ghanaians make this ideal impossible. Consequently, various district schemes charge flat rates for non-formal sector workers. By this arrangement, various district schemes charge premiums on the basis of economic potentials and activities prevailing at the district. Presently, the range of premiums is between GH¢7 and GH¢40 (approximately $3.5-$20.0). By June 2010, there were 145 schemes in operation across the country with a total registered membership of 15,555,815 (66.4%) of the population and 12,540,708 (53.6%) were active members of the scheme. Over 5,000 service providers were providing services on 95% of medical conditions. The scheme also has an exempt category of beneficiaries [informal adults aged ≥70 years, clients who are less than 18, expectant mothers, newly born babies and indigents] [21].

### Sources of data

Data for this paper are derived from the female data file of the 2008 Ghana Demographic and Health Survey (GDHS) collected by the Ghana Statistical Service under the wave of surveys supported by ICF Macro International. The surveys are largely carried out in developing countries where continuous population monitoring through civil registrations are inadequate. The dataset is the most recent conducted by the Ghana Statistical Service in collaboration with the Ghana Health Service and MEASURE DHS four years after the introduction of the NHIS. In the 2008 edition, 412 clusters were generated based on updated enumeration areas from the 2000 Population and Housing Census. Of the 412 clusters, about 12,000 households were included and 4,916 eligible women between the ages 15 and 49 years were subsequently interviewed, with a response rate of 97%. After recoding the data to suit our needs, the sample for this paper reduced to 4,910. The MEASURE DHS gave us the permission to use this data set following an assessment of a concept note on the paper. The data is publicly available (http://www.measuredhs.com).

### Analytical model

Because our interest was to examine the likelihood of insurance purchase, the outcome variable was coded 1 = “Yes” (insured) and 0 = “No” (uninsured). Since the outcome variable was a dichotomous variable, we employed a discrete choice model to measure how the various independent variables correlated with the outcome. Specifically, the logit function was employed since it allows estimation on a mixture of continuous and categorical variables. The choice of region of residence and wealth status or quintile was based on differences in poverty scores across the various regions. For instance, the Ghana Shared Growth and Development Agenda (2010–2013) recognise that there are inequities in health care access in the country, with the south benefiting more than the north. In order to explore interaction of household wealth status with region of residence, we created a spatial variable by collapsing the ten regions into three: Western, Central, Greater Accra, and Volta as the Coastal area/zone; the Eastern, Ashanti, and Brong-Ahafo were categorised as the Central/Middle zone and the Northern, Upper East and Upper West defined as the Northern zone. Household wealth status in DHS dataset is an aggregation through factor analysis of a number of household belongings such as car, agricultural land, refrigerator, bicycle, materials used in constructing housing, television, radio, type of household cooking fuel and a host of others [22]. The various levels of wealth status measurement in the dataset are poorest, poorer, middle/average, richer and richest.

At the first and second levels of inferential analysis, factorial interaction was applied to spatial location and wealth status. This was intended to determine locational and wealth effects on insurance purchase alone and whether the effects remain when the two are interacted. The interaction of wealth with spatial zone helped us to identify differences within and among groups. Survey weights, which are typical of nationally representative surveys, were factored into both descriptive and inferential analyses. The weights help to offset the problems of over and under sampling associated with national surveys. That the individual respondents are nested in households also gave us the impetus to estimate robust standard errors to account for non-independence of individuals selected from the same household. Other independent constructs which influence decisions towards insurance purchases such as individual and partner’s education, religion, and age were used to adjust for the effects of spatial location and wealth index or status. All the analyses were conducted with STATA, 12th version (College Station, Texas 77845 USA).

## Results

Table 1 presents respondents who were registered, broken down into various socioeconomic characteristics. Overall, there was less than half (40.19%) of the weighted sample that was covered under the scheme. While some of the independent variables showed significant association (95% CI) with insurance purchases, some did not. Variables that showed significant association with insurance subscription were region of residence, wealth status, education (individual and partner’s), age and religion. These give an indication of possible significant differences among the respective categories of the variables. It is again observed that less than half of women under the various socioeconomic characteristics had insurance, except among women whose partners had attained higher formal education and women who earned about the same as their partners as well as highly educated women. In respect of our main variables of interest (wealth and spatial location), the proportion of registered women under the scheme increased with increase in wealth status (Table 1). By spatial location, the highest proportion of women registered with the scheme was in the Central zone while the least was from the Coastal zone. Relative to age, respondents who are aged 40–44 had the highest propensity to purchase insurance. Personal health decision-making, household purchases (large and small) and the difference in couples’ earnings showed insignificant association with insurance purchases.

**Table 1:** Extent of insurance coverage by background characteristics

**Variable**	**Correlates**	**N = 4910**	**Proportion registered**
Wealth index^*^	Poorest	546	29.9
	Poorer	607	32.42
	Middle	601	38.49
	Richer	607	45.67
	Richest	573	49.44
Region^*^	Coastal	1509	29.86
	Central	967	49.53
	Northern	459	45.36
Respondent education^*^	None	699	32.68
	Primary	679	31.18
	Secondary	1477	45.06
	Higher	79	58.34
Age^*^	15-19	628	38.56
	20-24	571	34.78
	25-29	486	41.56
	30-34	366	43.34
	35-39	365	42.57
	40-44	258	44.99
	45-49	259	39.44
Religion^*^	Orthodox Christian	786	45.26
	Pentecostal/Other Christian	1430	39.64
	Moslem	444	39.82
	Traditional	159	22.38
	No religion	116	27.55
Health decision-making	Alone	406	44.21
	Respondent & partner	697	45.42
	Partner alone/Someone	542	41.40
Small household purchases	Alone	342	42.63
	Respondent & partner	666	45.69
	Partner alone/Someone	637	42.5
Large household purchases	Alone	733	42.56
	Respondent & partner	561	44.49
	Partner alone/Someone	351	39.89
Variations in couples’ earnings	More than partner	136	45.06
	Less than partner	938	45.06
	About the same	124	55.51
	Others	76	44.49
Partner’s education^*^	No education	600	30.22
	Primary	161	30.58
	Secondary	1054	44.18
	Higher	124	63.22

Chi-square: ^*^p < 0.05.

In Table 2, we present results of the binary logistic models of coverage of health insurance. Two models are presented here as follows: Model 1 examines the unadjusted effects of zone and wealth status; Model 2 focuses on the mixed effects of ecological zone and wealth index as well as other socioeconomic factors for calibration. In both models, however, the factorial interactions remain. The results portray significant differences in the likelihood of insurance subscription and spatial location as well as wealth status. For example, in Model 1 Table 2, the odds ratio of coverage was more than two folds in the Northern zone. By wealth status, women who were higher on the wealth gradient (richer/richest) were more than thrice likely to have purchased insurance.

**Table 2:** Binary logistic regression on health insurance ownership among women in Ghana

**Explanatory variables**	**Model 1**	**95% CI**	**Model 2**	**95% CI**
***Zones/Belts***				
Coastal	1	[1,1]	1	[1,1]
Central	1.761	[0.922-3.362]	1.355	[0.633-2.899]
Northern	2.318^**^	[1.328-4.045]	2.593^**^	[1.394-4.823]
***Wealth status***				
Poorest	1	[1,1]	1	[1,1]
Poorer	1.313	[0.802-2.150]	0.852	[0.490-1.483]
Middle	1.312	[0.767-2.244]	0.762	[0.416-1.395]
Richer	1.946^*^	[1.140-3.321]	1.215	[0.667-2.215]
Richest	3.029^***^	[1.787-5.136]	1.572	[0.858-2.880]
***Interactions – location & wealth status***				
Coastal with wealth (overall reference)	1	[1,1]	1	[1,1]
**Central interaction with wealth**				
Central # Poorest	1	[1,1]	1	[1,1]
Central # Poorer	1.240	[0.608-2.531]	1.697	[0.728-3.956]
Central # Middle	1.775	[0.857-3.674]	2.491^*^	[1.032-6.010]
Central # Richer	1.732	[0.833-3.602]	2.157	[0.890-5.228]
Central # Least poor	1.495	[0.702-3.185]	2.076	[0.812-5.307]
***Northern interaction with wealth***				
Northern # Poorest	1	[1,1]	1	[1,1]
Northern # Poorer	1.209	[0.640-2.283]	1.980	[0.894-4.383]
Northern # Middle	2.574^**^	[1.261-5.255]	4.597^***^	[1.906-11.09]
Northern # Richer	4.042^***^	[1.807-9.042]	8.475^**^	[2.174-33.03]
Northern # Richest	2.319	[0.970-5.545]	6.315^**^	[1.692-23.57]
***Respondent’s education***				
No formal education			1	[1,1]
Primary			0.897	[0.656-1.227]
Secondary			1.387^*^	[1.035-1.859]
Higher			1.255	[0.695-2.264]
***Religion***				
Catholic			1.332	[0.871-2.039]
Protestant			1.440	[0.968-2.141]
Pentecostal			1.326	[0.925-1.901]
Moslem			1	[1,1]
Traditional/No religion			0.737	[0.471-1.152]
***Age cohort***				
15-19			1	[1,1]
20-24			2.146	[0.851-5.411]
25-29			2.160	[0.862-5.414]
30-34			2.368	[0.934-6.004]
35-39			2.708^*^	[1.108-6.616]
40-44			2.942^*^	[1.182-7.325]
45-59			2.276	[0.889-5.827]
***Health decision making***				
Alone			1	[1,1]
Respondent & partner			1.111	[0.864-1.430]
Partner alone/Someone			1.023	[0.780-1.342]
***Small household purchases***				
Alone			1	[1,1]
Respondent & partner			1.093	[0.801-1.491]
Partner alone/Someone			1.153	[0.861-1.544]
***Large household purchase***				
Alone			1	[1,1]
Respondent & partner			0.953	[0.747-1.215]
Partner alone/Someone			1.067	[0.809-1.408]
***Variations in couples’ earnings***				
More than partner			1	[1,1]
Less than partner			1.010	[0.725-1.407]
About the same			1.062	[0.699-1.615]
Others			1.152	[0.684-1.943]
***Partner’s education***				
No education			1	[1,1]
Primary			1.108	[0.742-1.654]
Secondary			1.602^**^	[1.174-2.188]
Higher			2.458^***^	[1.542-3.919]
Cons	0.232^***^	[0.144-0.373]	0.0709^***^	[0.0232-0.217]
AIC	6226.5		2857.7	
N	4910		2288	

Exponentiated coefficients; 95% confidence intervals in brackets.

^*^p < 0.05 ^**^p < 0.01 ^***^p < 0.001.

**Figure 1:**
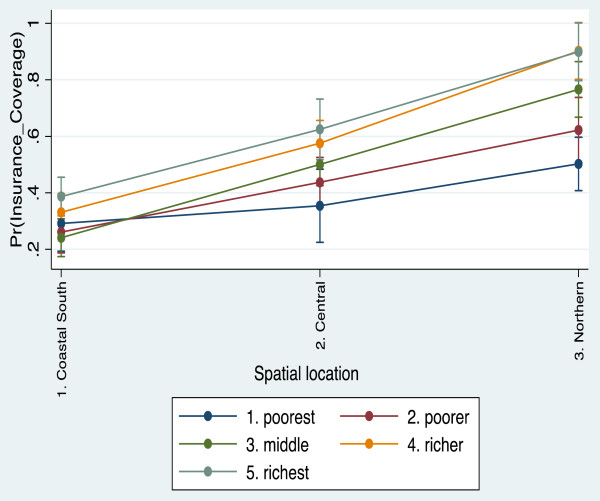
Predictive margins of insurance subscription by location and wealth.

Following the interaction of wealth with spatial zone, the significant differences still remain. The likelihood of purchasing insurance among women who are average and richer on the wealth gradient was about 150% - 300% higher. However, in Model 2 where we adjusted for the impacts of other socio-demographic variables, between group and within groups differences are illuminated better. In particular, the within group gap among the middle (OR = 4.6, CI 19–11.1), richer (OR = 8.5, CI 2.2-33.03) and richest (OR = 6.3, CI 1.7-23.6) populations on one hand and that of the poorer (OR = 1.98, CI 0.89-4.4) and the poorest (reference category) on the other in the Northern zone widens substantially. However, between group comparisons of the odds ratio for the poor and poorest in the Northern and Coastal zones, we find no significant differences, although the odds were higher in the Northern zone. Again, we also noticed higher likelihood of insurance subscription in the Northern areas compared to the Central areas. Figure 1 provides a graphical representation of predictive margins of interactive effects of spatial location and wealth status. Between the Coastal and Central zones, the odds were fairly constant by wealth quintile except in the Northern zone where the marginal effects are relatively pronounced.

Statistically significant impacts were also observed on age, personal education, and partner’s level of education. We also observed that partner’s education was more important predictor of health insurance subscription than individual’s own level of education. The likelihood of being a member of an insurance scheme was higher among women whose partners had obtained higher formal education (OR = 2.5, CI = 1.5-4.0) as compared to respondents who had attained higher education themselves (OR = 1.225, CI 0.7-2.3). Differences in religious affiliation are noted but not significant. In terms of likelihood, the non-Christian groups (Traditional believers and Moslems) were less likely to own health insurance. Among the various factors that are proxy for autonomy (autonomy in health decision making, household purchases whether large or small), there are no statistical differences.

## Discussion

In this paper, we examined the interactive effects of wealth status and spatial location (Coastal, Central and Northern) on ownership of health insurance among women. Data was drawn from the most recent women’s data file of Ghana Demographic and Health Survey (2008). Compared to most developing countries, Ghana is one of the few countries which have formally replaced out-of-pocket payment with insurance for health care delivery. Although the scheme has been described as one of the progressive schemes because of its indirect tax funding, it is, at the same time, considered regressive relative to informal sector contributions [23]. The study revealed somewhat significant socioeconomic and spatial differences in insurance subscription or purchase among Ghanaian women.

We noticed that there are significant differences in subscription by spatial location of respondents. Somehow, the differences in subscription appear to favour the rich in some parts of the country more than others. The differences we noticed are more pronounced between the poor and the rich in the Northern area. In a recent review of health care financing equity in three African countries, Mills et al. [23] indicated that the refusal of the poor to enrol in insurance schemes may be an indication of inequity since the poor indirectly pay towards funding insurance through indirect taxes. In Ghana, this may be the case given that a colossal proportion of insurance funds (about 60%) are obtained from the 2.5% health insurance levy taxed on certain goods and services.

In some respects, the findings are consistent with those made by Schneider [11], who found that poverty was a major reason against health insurance subscription. Several theoretical constructs can help put the findings in perspective. For instance, risk aversion, lack of trust in the system, high value for current consumption or simply inability to pay for existing premiums could account for the huge within group gaps in insurance purchase. Although the findings relative to spatial and wealth scores are aggregated, they provide useful pointers to the policy makers on areas to explore towards achieving equity. Evidence from Mexico’s PROGRESSA project has provided some useful lessons for analysis based on space and wealth scores, where geographic targeting was used as the first step to identify populations in need of interventions, followed by small area wealth matching to identify those in absolute need [24].

The results again point to class dimension in insurance purchase. Women whose partners are better educated and wealthier were more likely to have purchased health insurance compared to the less educated and poorer Ghanaians. These findings are in agreement with other studies (e.g. 6, 17, 25) on determinants of health insurance subscription. In a South African study, Kirigia et al. [25] noted that better education significantly accounted for being insured. The positive relationship between insurance and education in Ghana may be accounted for by employment opportunities and subsequent registration for health insurance. Better educated women and their partners are more likely to work in the formal sector. Under Ghana’s health insurance scheme, persons working in the formal sector do not make direct cash contribution to the scheme. Premiums among formal sector workers are deducted at source and dependents of formal sector employees pay abated premium. Perhaps, this might have accounted for the pattern observed. The fact that partner’s education predicts insurance subscription better than an individual’s own education points to the significant umpire roles men play in women’s health decision making. This finding is largely coherent with some preceding studies on influences of partners’ characteristics on healthy behaviours decision-making [26]. The observation in this study demonstrates the weight of intimate partners’ influences on health behaviours. This underscores the importance of appealing to partners’ as well as individuals’ beliefs in settings where male chauvinistic tendencies are high.

The likelihood of health insurance subscription increases with aging and this could be an indication of risk perception. Kirigia et al. [25] found direct effect of age on health insurance; thus aging positively predicts health insurance subscription. The positive connection between aging and health insurance purchasing has been attributed to biological degeneration in health, which makes older people appreciate the need to make extra investments in health, with health insurance being one principal focus.

## Conclusion

This study sought to investigate health insurance subscription among women in the reproductive ages, 15 – 49 years and we took a detour from previous studies to test the interactive effect of spatial location and wealth status on insurance subscription. At the interactive stage, significant differences within groups become clearer, observing that the poorest in the Northern zone were significantly less likely to have purchased insurance. This is notwithstanding the observation that women in the Northern zone were more likely to be insured than their counterparts in the Coastal and Central zones. The results underscore the relevance of geographic and proxy means targeting in identifying populations who may be in need of special interventions as part of the efforts to increase enrolment as well as means of social protection against the vulnerable.

## Competing interest

The author declares that they have no competing interest.

## Authors’ contribution

AKK and JAA conceptualized the study. JAA analysed the data and drafted the manuscript. All authors participated in the interpretation of data. All authors gave final approval of the version to be published.

## Pre-publication history

The pre-publication history for this paper can be accessed here:

http://www.biomedcentral.com/1472-6963/13/221/prepub
